# Supplementary material for: Simultaneous magnetic resonance imaging of pH, perfusion and renal filtration using hyperpolarized 13C-labelled Z-OMPD
Source: Nat Commun. 2023 Aug 21;14:5060. doi: 10.1038/s41467-023-40747-3 (PMC10442412; doi:10.1038/s41467-023-40747-3)
Supplement: Supplementary file 1 — Supplementary Information [file 41467_2023_40747_MOESM1_ESM.pdf]

Simultaneous Magnetic Resonance Imaging of pH, Perfusion and Renal Filtration using Hyperpolarized ^{13}C-labelled Z-OMPD

Martin Grashei*[1], Pascal Wodtke*[1], Jason G. Skinner[1], Sandra Sühnel[1], Nadine Setzer[1], Thomas Metzler[2], Sebastian Gulde[3], Mihyun Park[4], Daniela Witt[4], Hermine Mohr[3], Christian Hundshammer[1], Nicole Strittmatter[4], Natalia S. Pellegata[3,5], Katja Steiger[2], Franz Schilling[1,6,7]

Author affiliations.
[1]Department of Nuclear Medicine, TUM School of Medicine, Klinikum rechts der Isar, Technical University of Munich, D-81675 Munich, Germany
[2]Comparative Experimental Pathology (CEP), Institute of Pathology, School of Medicine, Technical University of Munich, D-81675 Munich, Germany
[3]Institute for Diabetes and Cancer, Helmholtz Zentrum München, D-85764 Neuherberg, Germany
[4]Department of Chemistry, TUM School of Natural Sciences, Technical University of Munich, D-81675 Munich, Germany
[5]Department of Biology and Biotechnology, University of Pavia, I-27100 Pavia, Italy
[6]Munich Institute of Biomedical Engineering, Technical University of Munich, D-85748 Garching, Germany
[7]German Cancer Consortium (DKTK), Partner Site Munich and German Cancer Research Center (DKFZ), D-69120 Heidelberg, Germany

*These authors contributed equally

Supplementary material

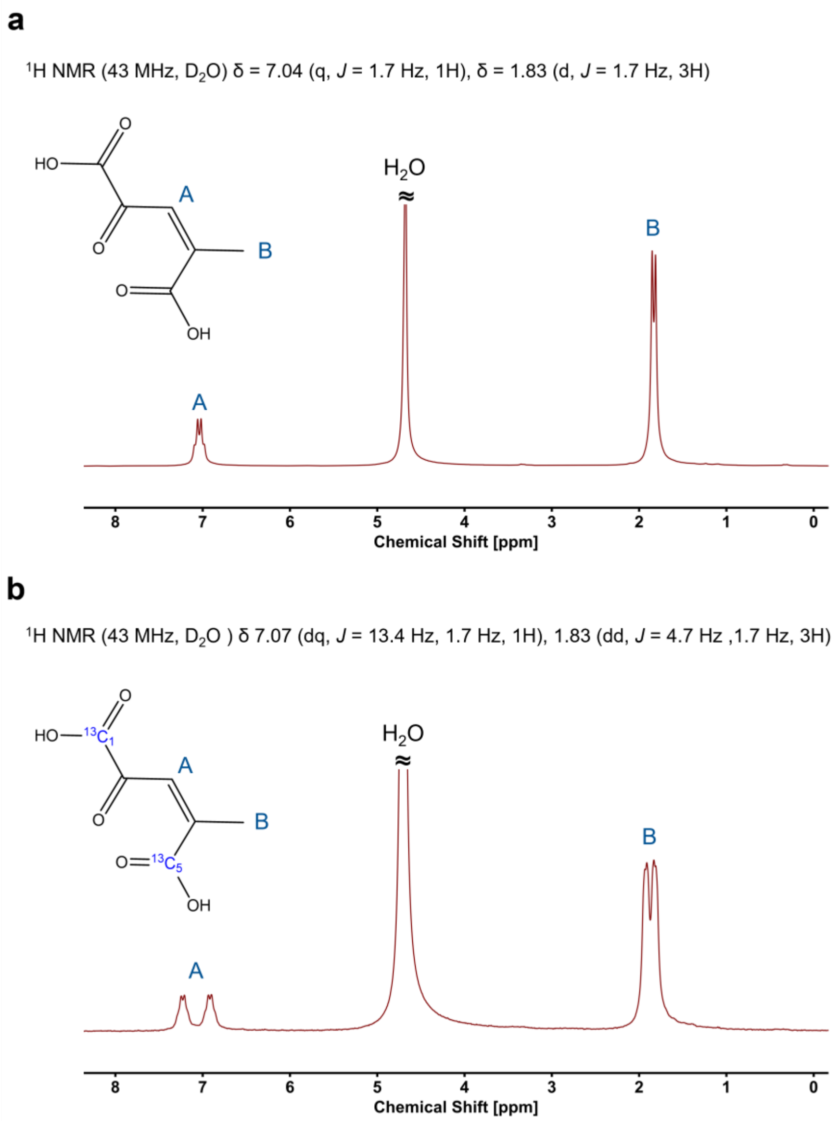

Supplementary Figure 1 | ^{1}H NMR spectra of the synthesized unlabelled Z-OMPD and ^{13}C-labelled [1,5-$^{13}C_2$]Z-OMPD. (a) Unlabelled Z-OMPD generates a quartet (A) and a doublet (B) with peak positions matching previous reports in literature[1,2]. **(b)** [1,5-$^{13}C_2$]Z-OMPD exhibits an additional splitting of both multiplets, generated by heteronuclear coupling between the $^{13}C_5$ nucleus and the corresponding protons. Both compounds were synthesized as described in the methods section and dissolved in D_2O for spectroscopy. Spectra were acquired at 1 T without water suppression.

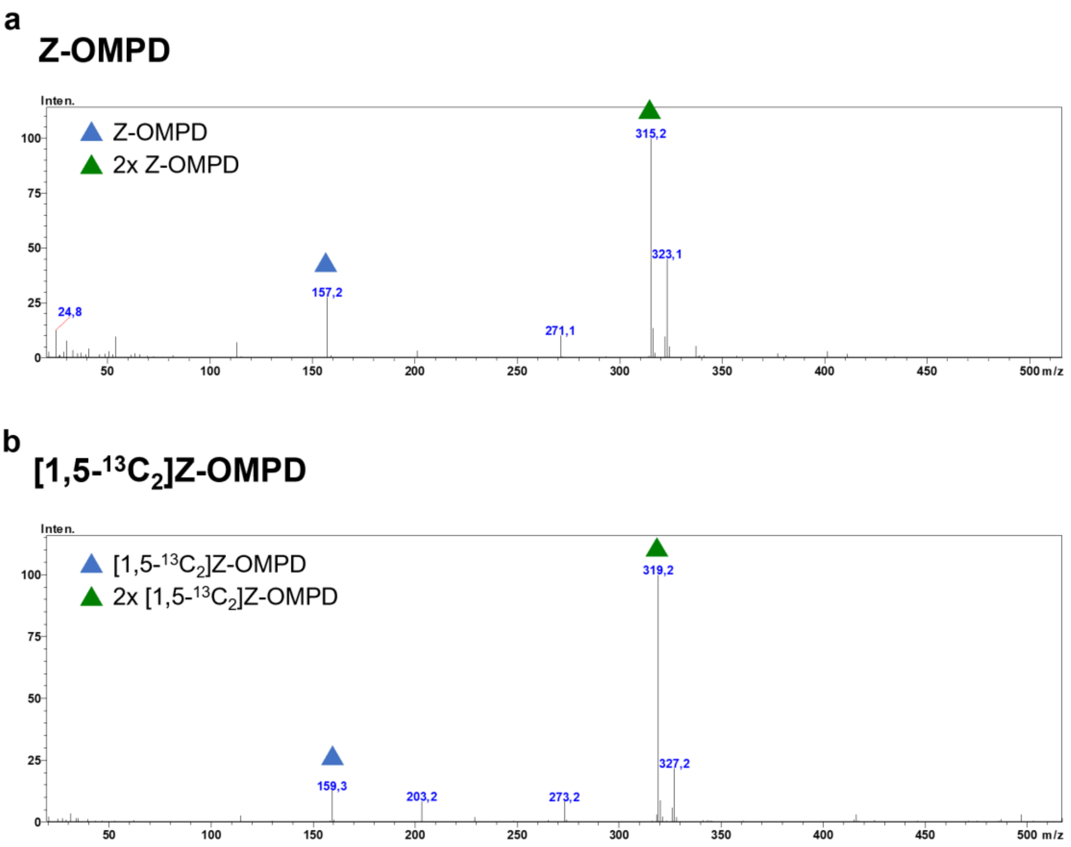

Supplementary Figure 2 | Negative mode mass spectra of unlabelled Z-OMPD and ^{13}C-labelled [1,5-$^{13}C_2$]Z-OMPD. (a) Unlabelled Z-OMPD has a mass m/z = 157.2 (blue triangle). The dominating peak in the spectrum is generated by a dimer of Z-OMPD at m/z = 315.2 (green triangle). **(b)** As expected, [1,5-$^{13}C_2$]Z-OMPD has a mass m/z = 159.3 g/mol, the spectrum is again dominated by the corresponding dimer m/z = 319.2.

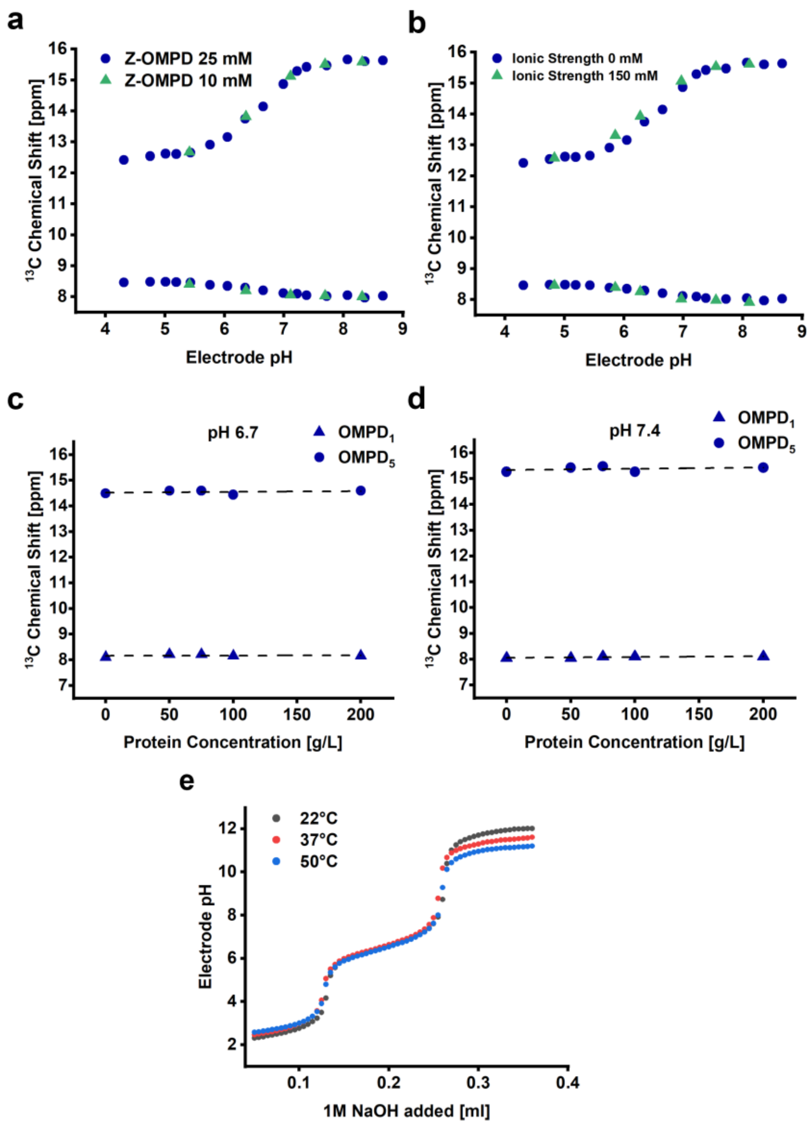

Supplementary Figure 3 | Influence of *in vivo* relevant environmental parameters on the pH sensitivity of Z-OMPD. (a) Titration curves recorded at [1,5-$^{13}C_2$]Z-OMPD concentrations of 10 mM and 25 mM show no *in vivo*-relevant influence of pH sensor concentration on pH sensitivity. **(b)** Titration curve acquired at 0 mM and 150 mM ionic strength (NaCl) show a slight shift of the $\Delta pK_a = -0.7 \cdot 10^{-3}$ pH/mM, translating into a pH uncertainty of 0.03 pH units in the *in vivo* relevant range of ionic strength[3]. **(c, d)** ^{13}C chemical shifts of the C_5- and C_1-resonance of [1,5-$^{13}C_2$]Z-OMPD at varying protein (bovine serum albumin) concentrations at pH 6.7 **(c)** and at pH 7.4 **(d)**. The maximum change in chemical shift extracted from linear fits is $4.7 \cdot 10^{-3}$ ppm/pc, (pc = protein concentration in g/L). With the physiological protein concentration range being 60 – 80 g/L[4], this translates into a pH uncertainty of 0.01 pH units. **(e)** Titration curves acquired at different temperatures to assess the impact of temperature on the pH sensitivity of Z-OMPD. No notable change of both pK_a-values can be observed across a large temperature range.

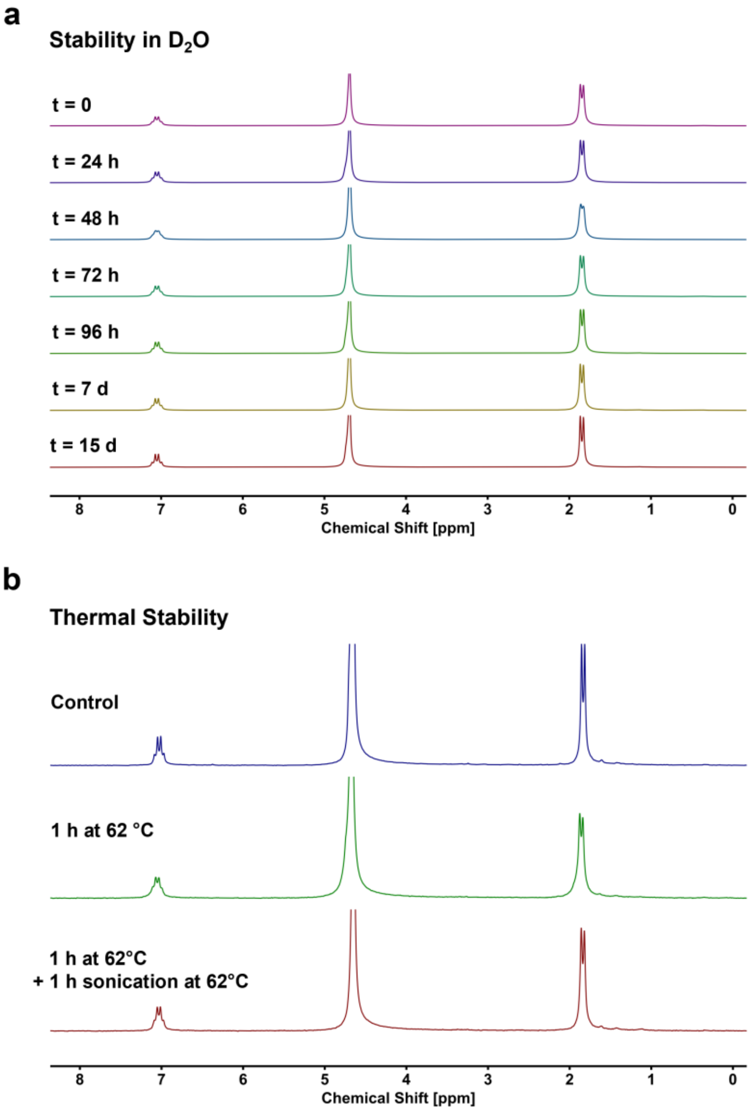

Supplementary Figure 4 | Chemical and thermal stability tests of Z-OMPD. (a) Z-OMPD was dissolved in D_2O and ^1H spectra were acquired over a period of 15 days. Over the entire time course, no degradation and respective decay products appear in the spectra, suggesting that Z-OMPD is chemically stable in D_2O for long time periods. **(b)** Thermal stability was tested by heating the sample to 62°C for one hour and subsequently sonicating it equally long at elevated temperature. Here, none of those influences appears to affect Z-OMPD's stability.

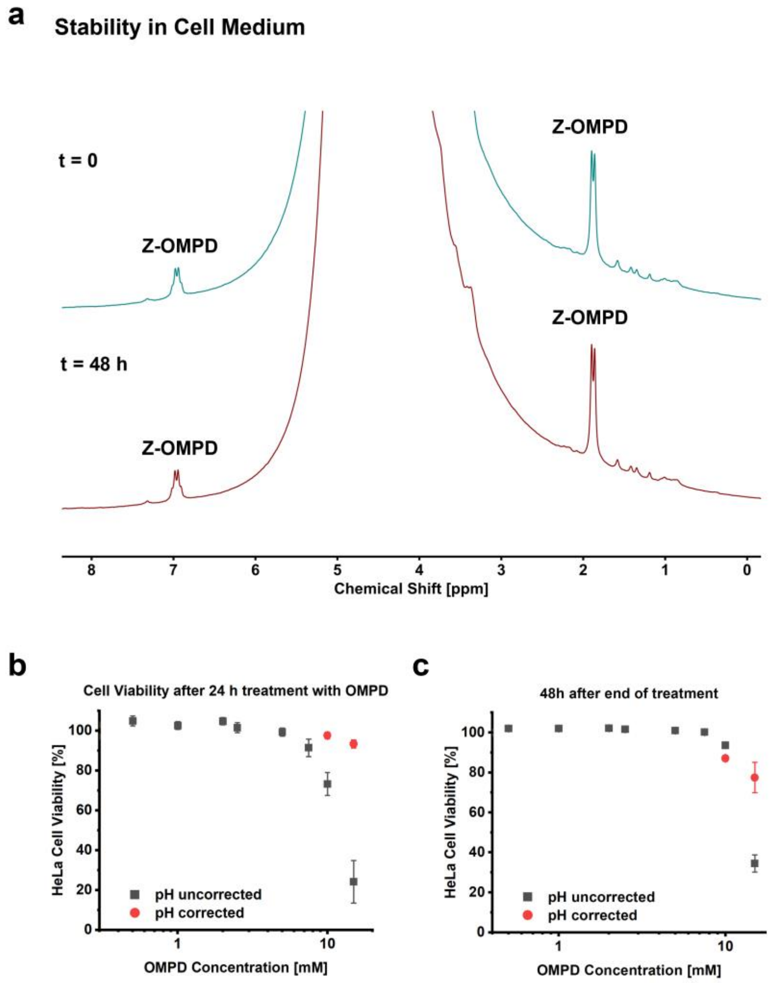

Supplementary Figure 5 | Assessment of cytotoxicity of Z-OMPD using Alamar Blue assays with HeLa cells. (a) To assure the validity of cytotoxicity assays, the stability of Z-OMPD in cell medium was checked using ^{1}H NMR to exclude unwanted influences of Z-OMPD on the cell medium and vice versa. **(b)** Varying concentrations (0 - 15 mM) of Z-OMPD were incubated with HeLa cells for 24 h and an Alamar Blue assay was performed directly after the incubation period. Due to gradual acidification of the cell medium by Z-OMPD, a reduction of cell viability can be observed at higher Z-OMPD concentrations. Further assays, for which the medium pH was adjusted prior to incubation, resulted in no notable reduction of cell viability at the highest concentrations. **(c)** To further assess slow toxicity processes, cells were incubated for 24 h with Z-OMPD and incubated another 48 h without Z-OMPD after which an Alamar Blue assay was performed. Again, a pH-related reduction in cell viability can be seen at high Z-OMPD concentrations which diminishes upon medium pH adjustment prior to incubation. Overall, no indication for concerning cytotoxicity of Z-OMPD was found. Values are presented as means ± SD (*n* = 3 individual cell samples).

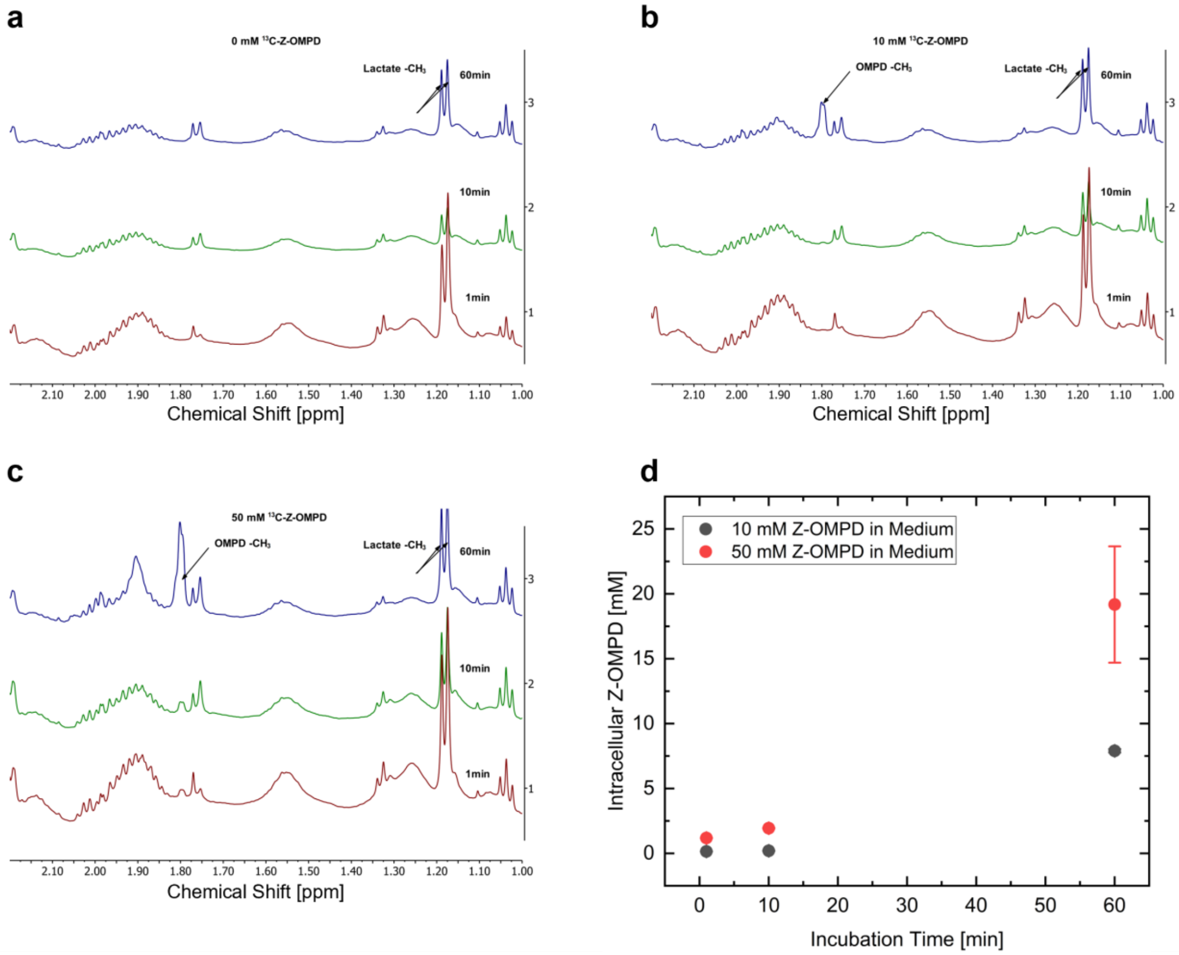

Supplementary Figure 6 | Assessment of intracellular uptake of Z-OMPD. (a) Control ^{1}H NMR spectra of EL4 cell lysates after varying incubation times. **(b)** ^{1}H NMR spectra of EL4 cell lysates after varying incubation times with cell medium containing 10 mM Z-OMPD. A duplet with increasing intensity for longer incubation times can be observed at 1.80 ppm, which corresponds to the methyl group of Z-OMPD and indicates increasing cellular uptake over time. **(c)** ^{1}H NMR spectra of EL4 cell lysates incubated with 50 mM Z-OMPD-containing medium. Higher Z-OMPD peaks with dynamics similar to the spectra series in **(c)** can be observed. **(d)** Back-calculated intracellular Z-OMPD concentration as a function of incubation time and surrounding Z-OMPD concentration. Significant cellular uptake over the time course of one hour can observed for both medium concentrations. This strong uptake after one hour suggests existence of suitable transporters for Z-OMPD uptake into the cell. However, the fraction of intracellular Z-OMPD remains less than 3% of the extracellular concentration on time scales relevant to hyperpolarized *in vivo* imaging (1 min). thereby validating Z-OMPD as an extracellular pH sensor for hyperpolarized MRI. Values are presented as means ± SD (*n* = 2 individual cell samples).

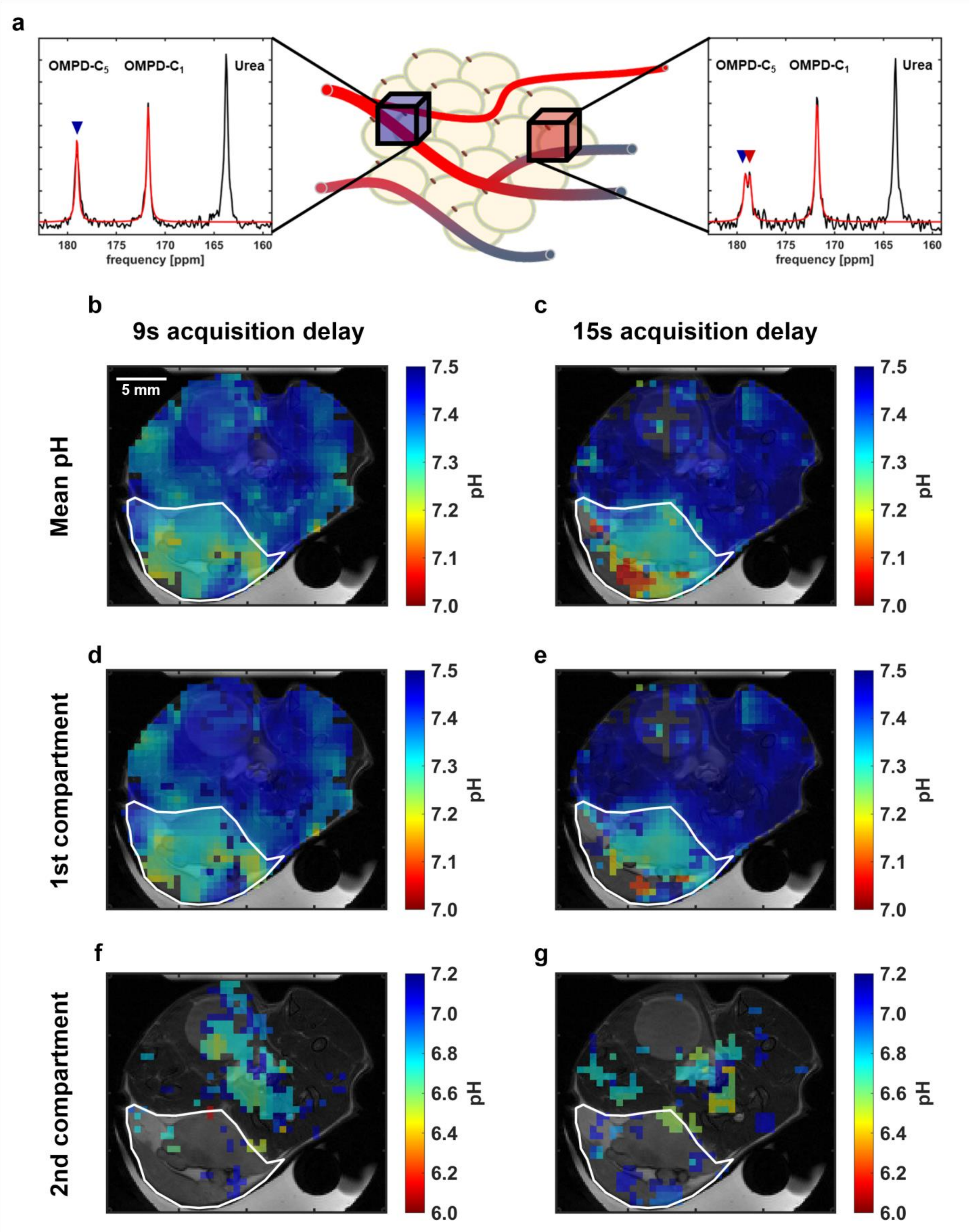

Supplementary Figure 7 | Temporal dependence of compartment detection and mean pH. (a) The observation of two pH compartments within tumor tissue can be explained by the pH sensor being located

either in vascular or extracellular space. For voxels, containing predominantly vessel signal, only one compartment can be observed (left spectrum), while voxels, covering also acidified extracellular space with sufficient tracer accumulation, a second peak, representing this tissue compartment can be observed (right spectrum). As the mean pH is weighted by the signal intensities from both observed compartments, delayed accumulation in the extracellular space compared to the fast perfused vascular space results in acquisition delay dependent mean pH contrast **(b, c)**. The physiological compartment **(d, e)** is present at both acquisition timepoints (9 s and 15 s post end of injection) due to the rapid perfusion whereas Z-OMPD is detectable on more tumor areas with an acidified extracellular space for later time points **(f, g)**. Here, increasing extravasation of Z-OMPD to the extracellular space with increasing acquisition delay competes with hyperpolarized signal decay for optimized imaging time points. Nevertheless, pH values for each compartment (**d-g**) agree with each other for both acquisitions, making pH compartment images quantitatively more robust with respect to timing variations compared to mean pH maps.

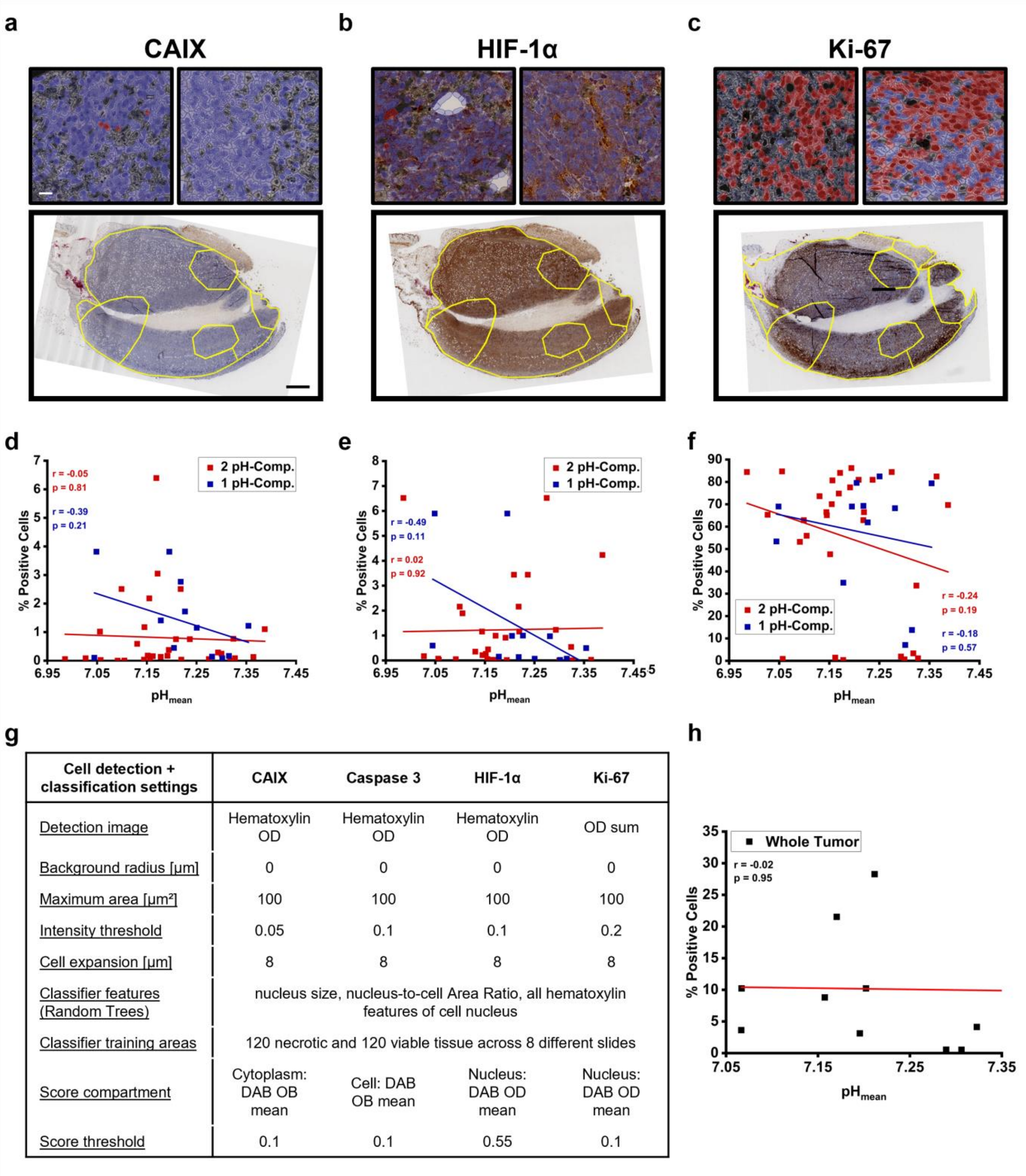

Cell detection + classification settings	CAIX	Caspase 3	HIF-1α	Ki-67
Detection image	Hematoxylin OD	Hematoxylin OD	Hematoxylin OD	OD sum
Background radius [μm]	0	0	0	0
Maximum area [μm²]	100	100	100	100
Intensity threshold	0.05	0.1	0.1	0.2
Cell expansion [μm]	8	8	8	8
Classifier features (Random Trees)	nucleus size, nucleus-to-cell Area Ratio, all hematoxylin features of cell nucleus			
Classifier training areas	120 necrotic and 120 viable tissue across 8 different slides			
Score compartment	Cytoplasm: DAB OB mean	Cell: DAB OB mean	Nucleus: DAB OD mean	Nucleus: DAB OD mean
Score threshold	0.1	0.1	0.55	0.1

Supplementary Figure 8 | Quantitative analysis of immunohistochemical stainings for correlation of histology and pH imaging. (a) EL4 tumor stained for CAIX. Overall, the tumor shows essentially no expression of this pH regulator protein (*n* = 9 tumors). White scale bar top left, 20 μm; black scale bar bottom, 1 mm. **(b)** While EL4 tumors overall show strong cytoplasmic positivity for HIF-1α (*n* = 9 tumors), the DAB stain did not locate in the nucleus and was therefore no specific for hypoxia. **(c)** Staining for Ki-67 shows strong positivity (*n* = 7 tumors) in viable tumor areas, confirming the strong proliferative potential of this fast-growing tumor. Zoomed regions top left: Representative area of strong positivity, top right: Representative area of low positivity. Red cells = classified positive; blue cells = classified negative; black

cells = necrotic, not included for analysis. Yellow ROIs in whole slide images indicate regions, where an acidified pH compartment was detected by hyperpolarized pH imaging (Fig. 4 c). **(d)** Neither the pH of areas bearing one nor the pH of areas bearing two pH compartments shows any correlation with the generally low expression level of CAIX. **(e)** With EL4 tumors only having low hypoxic fractions (HIF-1α positivity), no correlation with pH for both tumor area types can be observed. **(f)** Overall strong expression of Ki-67 appears to be uncorrelated to the tumor pH for areas either bearing either one or two pH compartments. **(g)** QuPath parameters for first-level cell detection and training of a random trees classifier for second-level classification of necrosis and viable tumor cells. For each staining, cell detection and classifier training was optimized separately. Viable cells were classified either as positive or negative based on staining-specific score compartments and optical density thresholds for the DAB stain channel. The entire histology analysis workflow was verified by a veterinary pathologist. **(h)** Mean pH values and fraction of caspase 3-positive cells averaged for whole tumors show no correlation for ten measurements in nine mice.

a
b
c
d
e

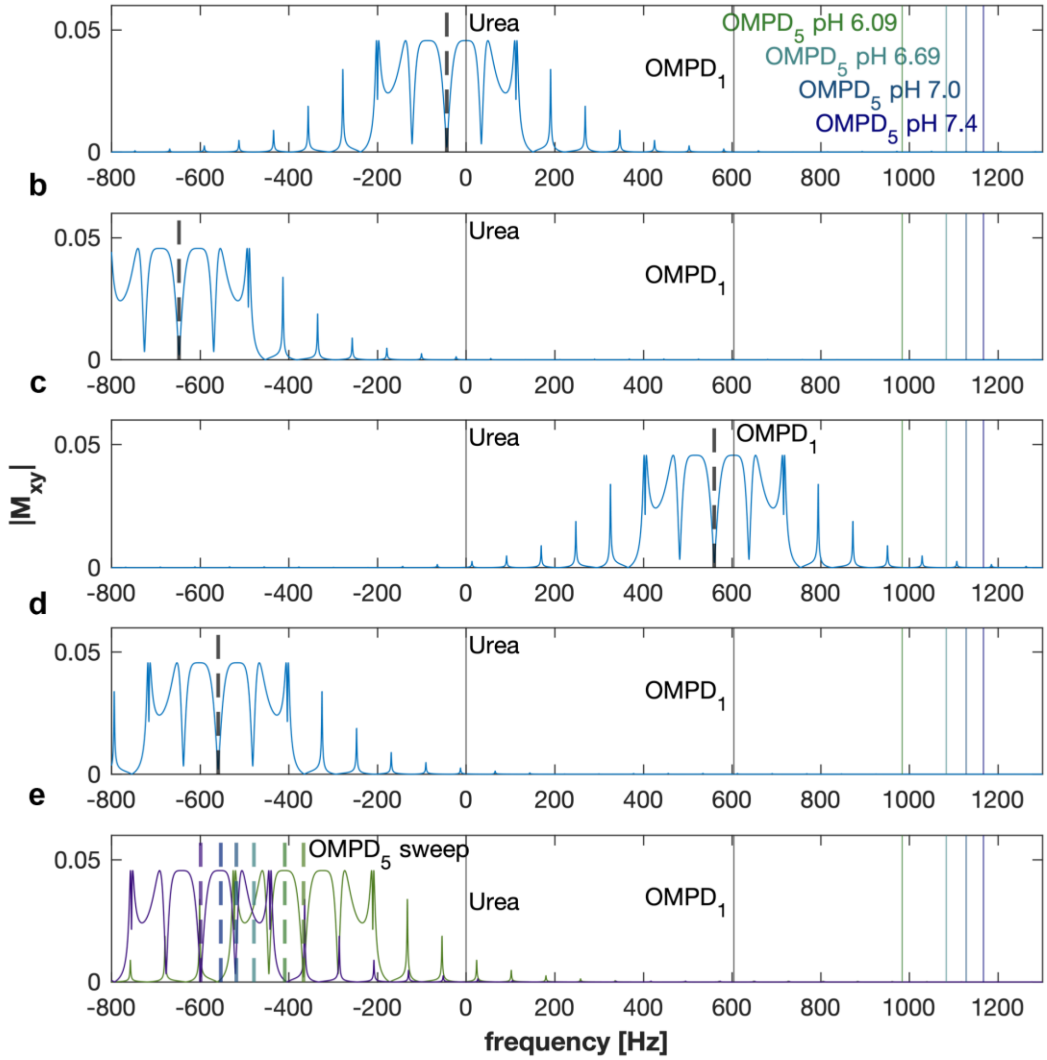

Supplementary Figure 9 | Balanced steady-state free precession response profiles for alternating, selective 3D imaging of ^{13}C-urea and Z-OMPD-C$_1$. Simulated bSSFP response profiles for co-polarized imaging experiments to selectively excite ^{13}C-urea **(a)** and Z-OMPD **(c)**. Additional response profiles are simulated for validation experiments **(b, d, e)** in which the contribution of non-target resonances to the target metabolite images are measured. Excitation with the profile in **(b)** simulates the contribution of Z-OMPD-C$_1$ to urea images, **(d)** simulates the contribution of ^{13}C-urea to Z-OMPD-C$_1$-images, and **(e)** simulates the contribution of Z-OMPD-C$_5$-signal to Z-OMPD-C$_1$-images. As the C$_5$-position shifts with pH, this was assessed for various pH scenarios with a frequency sweep of 6 images corresponding to a large range of pH values (5.5 to 7.5). Only response profiles for the highest (purple) and lowest pH values (green) are plotted for simplicity. Excitation frequencies of the RF pulses for the 6 images are indicated by the vertical dashed lines. For actual experiments, ^{13}C-urea was used as a model for OMPD-C$_1$ **(d)** and Z-OMPD-C$_5$ **(e)** in contamination experiments **(b, d, e)** due to proximity of the Z-OMPD-C$_5$-resonance to Z-OMPD-C$_1$. Simulations predict negligible contribution of all possible non-target resonances to the target resonance images. Simulation parameters: T_1 = 30 s, T_2 = 250 ms, TR = 12.8 ms, $\boldsymbol{\alpha}$ = 12°.

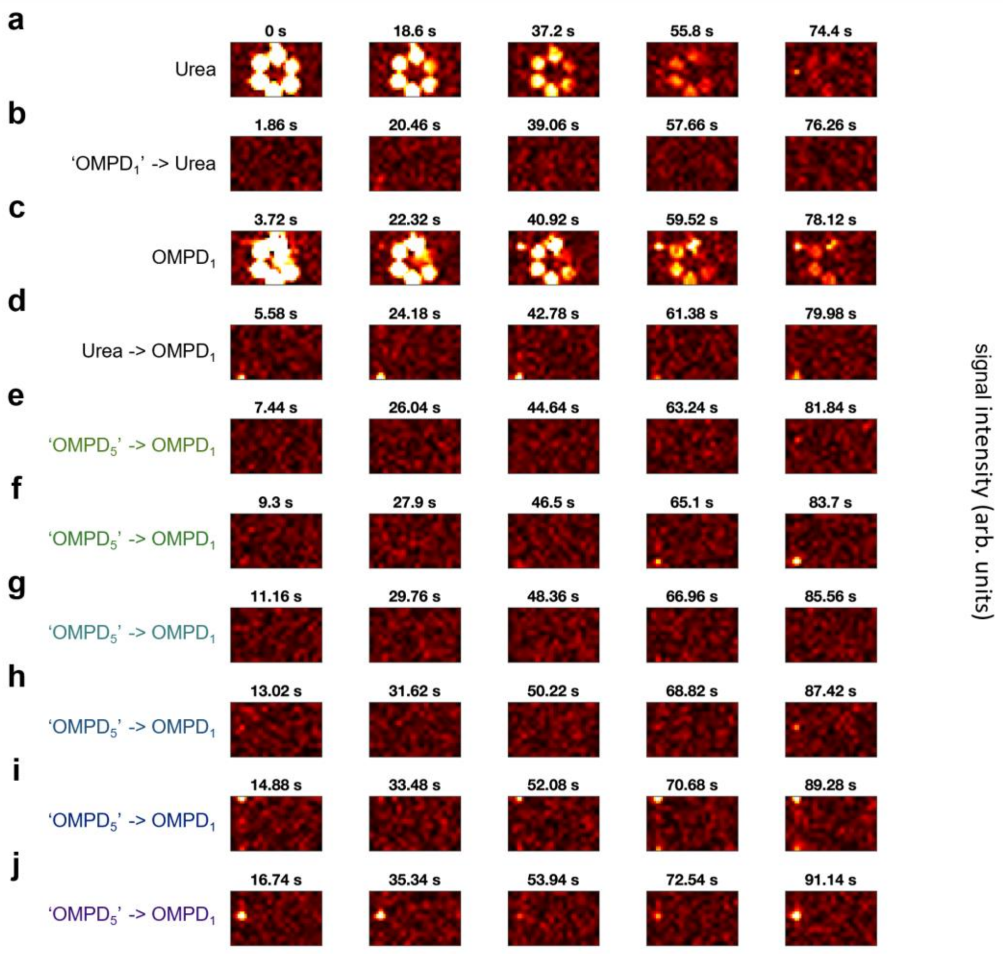

Supplementary Figure 10 | Validation of resonance-selectivity for bSSFP-based imaging of ^{13}C-urea and Z-OMPD-C_1. Validation images from experiments for selective imaging co-polarized ^{13}C-urea **(a)** and Z-OMPD-C_1 **(c)** in which the contribution of non-target resonances to the target images are measured **(b, d, e-j)**. **(b)** shows potentially signal of Z-OMPD-C_1 contaminating the ^{13}C-urea images. **(d)** represents the contamination signal ^{13}C-urea to Z-OMPD-C_1, and **(e)** that of Z-OMPD-C_5 to Z-OMPD-C_1-images. This was assessed in detail with a frequency sweep of 6 images corresponding to possible C_5-contaminations at a range of pH values (5.5 to 7.5). ^{13}C-urea was used as a dummy resonance for Z-OMPD-C_1 **(d)** and Z-OMPD-C_5 **(e-j)** in contamination experiments due to proximity of the Z-OMPD-C_5-resonance on the offside of Z-OMPD-C_1. All images together demonstrate that the bSSFP sequence selectively excites the target resonances ^{13}C-urea **(a)** and Z-OMPD-C_1 **(c)** in a very clean way with no contaminations from each other **(b, d)** or from the $OMPD_5$ resonance **(e-j)**, as predicted by the simulations (Fig. S8). Displayed are representative slides from a 1.8 s 3D spectrally selective bSSFP acquisition. TR=12.8 s, matrix=20x12x12, FOV = 60x36x36 mm^3, $\boldsymbol{\alpha}$ = 12°, RF excitation full width at half-max (FWHM) = 180 Hz.

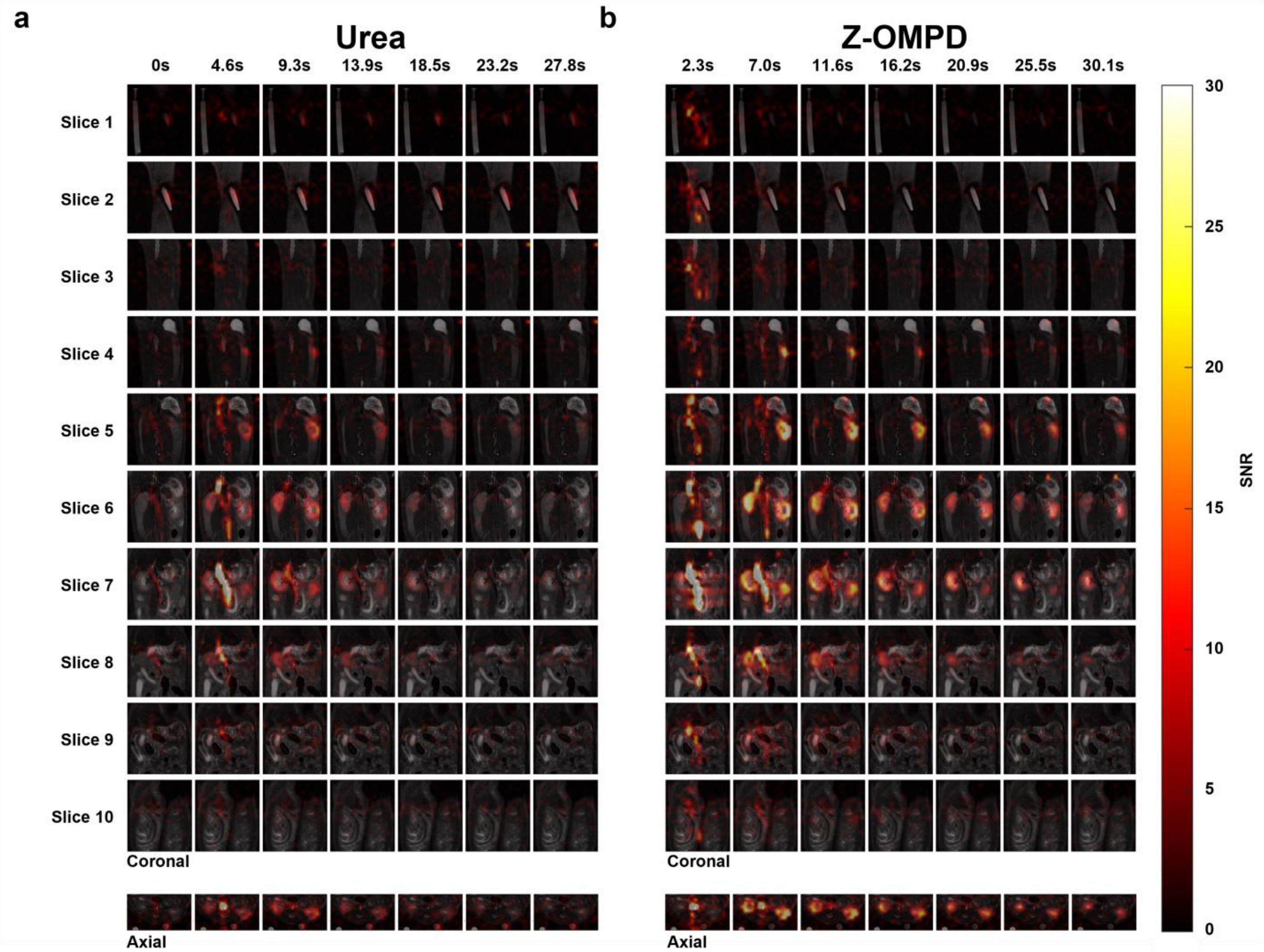

Supplementary Figure 11 | Full 3D image series from an alternating, selective bSSFP acquisition. Full three-dimensional image series of a bSSFP acquisition on a healthy rat kidney upon injection of co-polarized 13C-urea and [1,5-$^{13}C_2$]Z-OMPD. While the acquisitions cover the entire abdominal region of the kidney in dorsoventral direction, the signals for ^{13}C-urea **(a)** and Z-OMPD-C_1 **(b)** are well confined to the central vessels and the kidneys with no suspicious artifacts in any slice at any time point. Isotropic 3D acquisitions also allow rendering of corresponding axial images of both compounds in the kidneys (bottom rows).

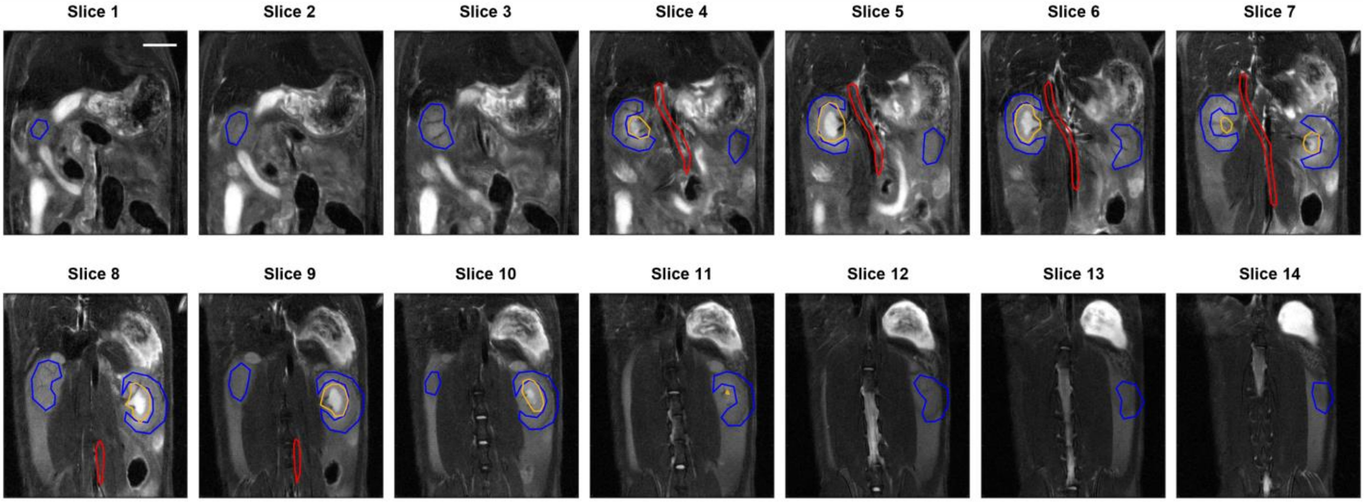

Supplementary Figure 12 | 3D region-of-interest placement for analysis of renal perfusion and kidney filtration function. High resolution anatomical T_2-weighted images on which three-dimensional regions-of-interest were placed for extraction of signal time curves for the central blood vessel (red) and the renal cortex (blue), which forms a half-sphere around the renal pelvis (yellow) which is located in the kidney center. Scale bar, 10 mm.

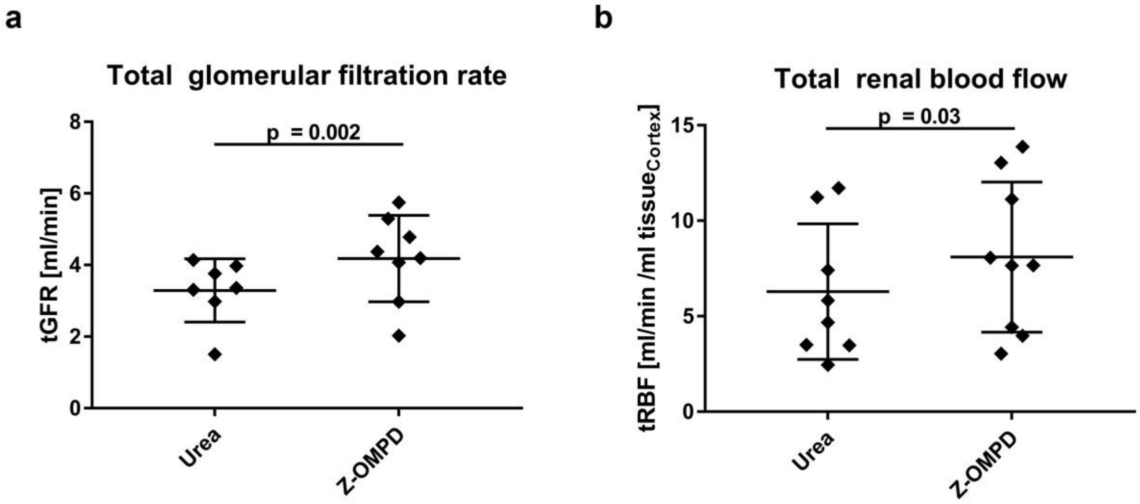

Supplementary Figure 13 | Comparison of cumulated glomerular filtration rates and renal blood flow for hyperpolarized [^{13}C]urea and [1,5-$^{13}C_2$]Z-OMPD. Summation of glomerular filtration rates and renal blood flow from both kidneys yields **(a)** total glomerular filtration rates (tGFR) and **(b)** total renal blood flow values (tRBF). tGFR measured by Z-OMPD appear to be significantly higher compared to Z-OMPD. This observation holds for tRBF values, however with the difference between urea and Z-OMPD being less prominent compared to tGFR. Values are presented as means ± SD. Two-tailed, unpaired Student's *t* tests were used (**a**: n = 7 [^{13}C]urea- and n = 8 Z-OMPD-based measurements in individual kidney pairs, n = 8 [^{13}C]urea- and n = 9 Z-OMPD-based measurements in individual kidney pairs).

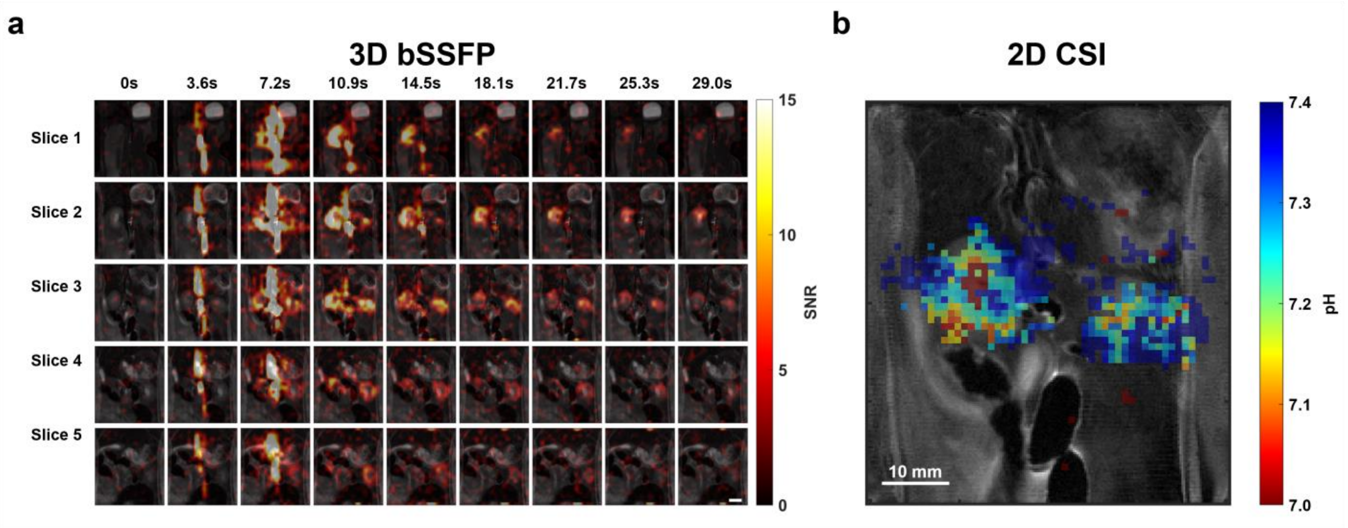

Supplementary Figure 14 | Simultaneous imaging of perfusion and pH using only hyperpolarized [1,5-$^{13}C_2$]Z-OMPD. (a) While co-injected, hyperpolarized ^{13}C-urea was used throughout this study as a chemical shift reference and reference perfusion marker, comparable perfusion characteristics and internal referencing for pH mapping allow simultaneous imaging of perfusion and pH with [1,5-$^{13}C_2$]Z-OMPD only. Compared to a co-injection protocol, a faster bSSFP imaging protocol allows acquisition of more time frames to measure perfusion within the first 30 seconds **(a)**, while a subsequent CSI acquisition allows mapping of renal pH **(b)** using C_1-resonance-residuals as internal reference for pH calculation. This simplifies the applicability of [1,5-$^{13}C_2$]Z-OMPD as co-polarizations can be omitted.

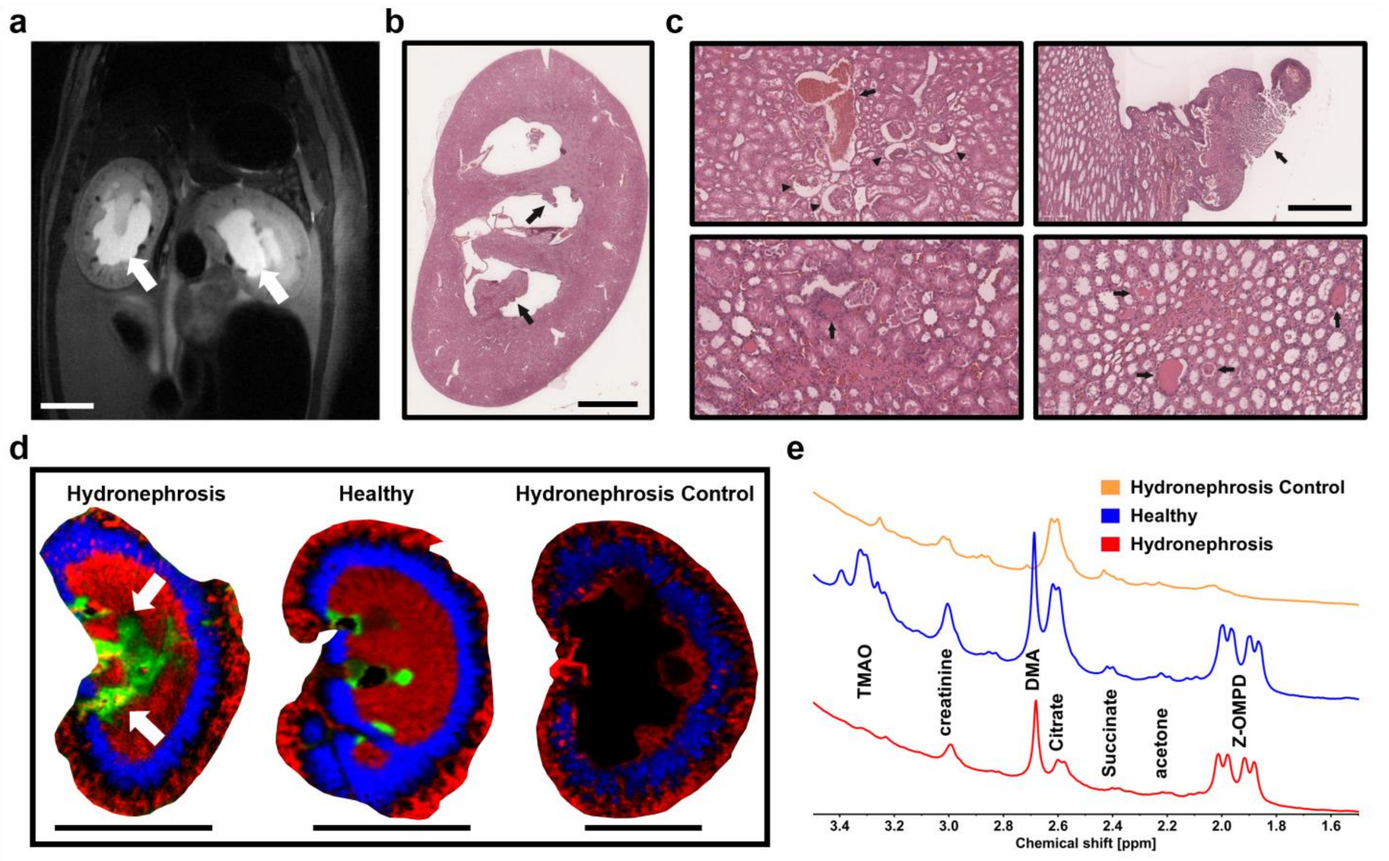

Supplementary Figure 15 | Characterization of hydronephrosis caused by pheochromocytoma in MENX rats. (a) Kidneys of rats developing gene mutation-induced pheochromocytoma exhibit abnormal morphology of the renal pelvis and medulla (*n* = 9 rats), together with dilatated ureters (white arrows) in anatomical T_2-weighted MRI images (n = , scale bar 10 mm. **(b)** H&E stains of resected kidneys confirm a dilated pelvis and atypical nodular urothelial hyperplasia (black arrows), scale bar 5 mm. **(c)** Further histopathological assessment reveals: (top left) dilated blood vessels (black arrow) and glomerular capsules with occasional filtration of erythrocytes (arrowheads), (top right), atypical nodular urothelial hyperplasia with ulceration and inflammatory infiltrates, (bottom left) early mineralization of proteinaceous fluid in the tubuli and (bottom right) tubuli filled with proteinaceous acellular fluid, scale bar 200 µm. **(d)** Residual kidney function and tracer filtration and excretion were confirmed by mass spectrometry imaging of resected kidneys 80 minutes post injection. Compared to HP ^{13}C-MRI, Z-OMPD is predominantly located to the renal pelvis of hydronephrotic kidneys (left) but at larger amounts compared to injected healthy controls (middle). Specificity of m/z of [1,5-$^{13}C_2$]Z-OMPD was confirmed with non-injected hydronephrotic controls (right). Molecules were tentatively assigned according to: green/probe: m/z = 159.02 (Z-OMPD, $[M-H]^-$), red (unspecific tissue for visual guidance): m/z = 306.08 (GSH, $[M-H]^-$), blue (visual guidance for cortex/medulla): m/z = 307.15; scale bar, 10 mm. **(e)** ^{1}H NMR spectroscopy indicates that Z-OMPD (~ 1.9 ppm) can be found in the urine after 80 minutes and confirms incomplete urinary tract obstruction. Filtered amount of Z-OMPD and also citrate is lower compared to injected healthy controls after equivalent time intervals.

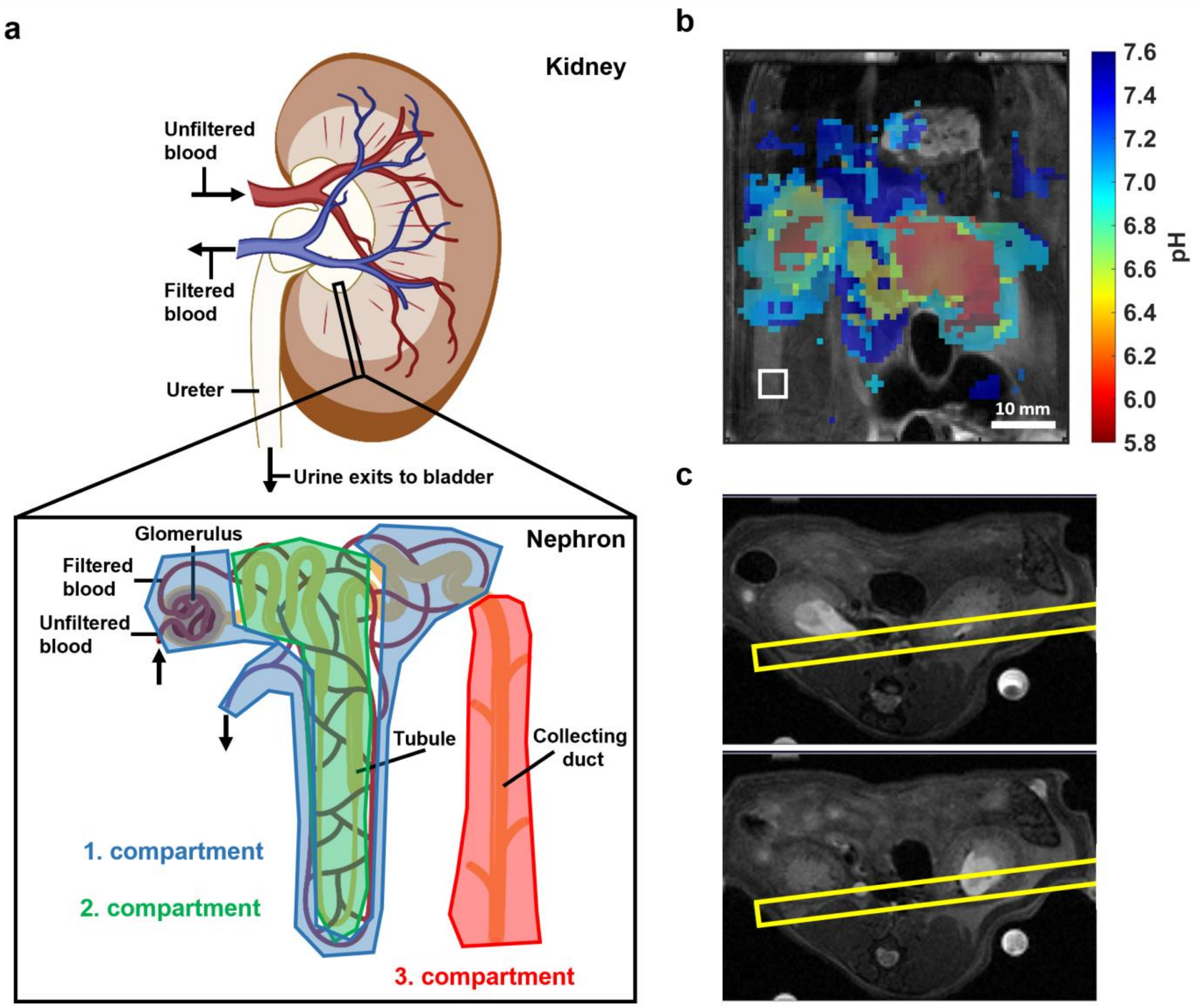

Supplementary Figure 16 | Renal pH compartment localization. (a) pH compartments detected by pH imaging can be localized to anatomical segments of the kidney (schematic figure includes a schematic kidney and schematic nephron which were created with BioRender.com, Toronto, Canada, license). Being a well-perfused and complex-structured, large amounts of blood are directed the renal cortex (**a**, left). As the cortex is therefore the best perfused area of the kidney, its pH is general very similar to the blood pH. This is reflected by the first pH compartment map (Fig. 7d), which mainly contains signal from blood vessels and the cortex. Since the entire kidney contains either renal blood vessels or cortex regions, the presence of this first compartment (Fig. 7d) across the entire kidney is well in line with anatomical structure. Its nephrons extend from the cortex through the medulla to the pelvis (**a**, bottom), where gradual acidification of the filtrate occurs[5]. Here, the glomeruli and the loop of Henle-surrounding vessels, with the latter extending throughout the entire medulla, also contain blood which also explains why the first pH compartment (Fig. 7d) covers renal cortex and medulla regions (**a**, bottom, blue-shaded area). Z-OMPD which enters the proximal tubules and potentially residues longest in the loop of Henle forms the second pH compartment (**a**, bottom, green-shaded area), which shows slight acidification due to the filtration-related acidification. These tubules start in the cortex and with the loop of Henle reaching far down into the medulla, this explains why the second pH compartment also covers most of the kidney (Fig. 7f). The third and most acidic pH compartment is assignable to the collecting duct and the renal pelvis (**a**, bottom, red-shaded area), which also starts upper region of the medulla and extends down to the renal pelvis. As the collecting duct and the renal pelvis also cover significant areas of the kidney, this explains why also the third pH compartment shows considerable

extension across the entire kidney (Fig. 7h). (**b**) Nevertheless, to highlight, that there is some slight discrimination between the spatial coverage of the pH compartments, we overlaid the compartments from Fig. 7d, f, h) in **b** of this section, which shows that the third compartment is slightly more confined to the center of the kidney, thereby matching the region assignments in **a**. The white square indicates the native CSI voxel size. **(c)** The used FIDCSI sequence is a 2D slice acquisition with a thickness of 4 mm (yellow box). As the respective model develops pheochromocytoma, capturing of the adrenal glands in addition to the kidneys was intended and positioning of the CSI slices was often a trade-off between coverage of all four organs (left kidney, right kidney, left adrenal gland, right adrenal gland) and optimal coverage of the kidneys. This often led to more coverage of volume for one kidney compared to the other. In some animals, for left kidney, this led to a much smaller fraction of the renal pelvis region being covered compared to the medulla and cortex, leading to the minor appearance of the respective pH compartment in Fig. 7h in the manuscript.

SUPPLEMENTARY REFERENCES

1 Ohyama, T., Hoshino, T. & Ikarashi, T. Isolation and structure of a new organic acid accumulated in tulip plant (Tulipa gesneriand). *Soil Science and Plant Nutrition* **34**, 75-86, doi:10.1080/00380768.1988.10415581 (1988).

2 Rios, A. C., Bera, P. P., Moreno, J. A. & Cooper, G. The Pyruvate Aldol Condensation Product. A Metabolite that Escaped Synthetic Preparation for Over A Century. *ACS Omega* **5**, 15063-15068, doi:10.1021/acsomega.0c00877 (2020).

3 Reynolds, R. M., Padfield, P. L. & Seckl, J. R. Disorders of sodium balance. *British Medical Journal* **332**, 702-705, doi:10.1136/bmj.332.7543.702 (2006).

4 Busher, J. T. in *Clinical Methods: The History, Physical, and Laboratory Examinations.* Ch. 101, (Butterworths, 1990).

5 Gaohua, L., Miao, X. & Dou, L. Crosstalk of physiological pH and chemical pKa under the umbrella of physiologically based pharmacokinetic modeling of drug absorption, distribution, metabolism, excretion, and toxicity. *Expert Opin Drug Metab Toxicol* **17**, 1103-1124, doi:10.1080/17425255.2021.1951223 (2021).
