# Supplementary material for: Simultaneous magnetic resonance imaging of pH, perfusion and renal filtration using hyperpolarized 13C-labelled Z-OMPD
Source: Nat Commun. 2023 Aug 21;14:5060. doi: 10.1038/s41467-023-40747-3 (PMC10442412; doi:10.1038/s41467-023-40747-3)
Supplement: Supplementary file 3 — Reporting Summary [file 41467_2023_40747_MOESM3_ESM.pdf]

nature portfolio

Corresponding author(s): Franz Schilling

Last updated by author(s): Jul 21, 2023

Reporting Summary

Nature Portfolio wishes to improve the reproducibility of the work that we publish. This form provides structure for consistency and transparency in reporting. For further information on Nature Portfolio policies, see our Editorial Policies and the Editorial Policy Checklist.

Statistics

For all statistical analyses, confirm that the following items are present in the figure legend, table legend, main text, or Methods section.

n/a	Confirmed	
☐	☒	The exact sample size (*n*) for each experimental group/condition, given as a discrete number and unit of measurement
☒	☐	A statement on whether measurements were taken from distinct samples or whether the same sample was measured repeatedly
☐	☒	The statistical test(s) used AND whether they are one- or two-sided<br>*Only common tests should be described solely by name; describe more complex techniques in the Methods section.*
☒	☐	A description of all covariates tested
☒	☐	A description of any assumptions or corrections, such as tests of normality and adjustment for multiple comparisons
☐	☒	A full description of the statistical parameters including central tendency (e.g. means) or other basic estimates (e.g. regression coefficient) AND variation (e.g. standard deviation) or associated estimates of uncertainty (e.g. confidence intervals)
☐	☒	For null hypothesis testing, the test statistic (e.g. *F*, *t*, *r*) with confidence intervals, effect sizes, degrees of freedom and *P* value noted<br>*Give P values as exact values whenever suitable.*
☒	☐	For Bayesian analysis, information on the choice of priors and Markov chain Monte Carlo settings
☒	☐	For hierarchical and complex designs, identification of the appropriate level for tests and full reporting of outcomes
☐	☒	Estimates of effect sizes (e.g. Cohen's *d*, Pearson's *r*), indicating how they were calculated

Our web collection on statistics for biologists contains articles on many of the points above.

Software and code

Policy information about availability of computer code

Data collection	MRI data acquisition: ParaVision 6.0.1 (Bruker BioSpin MRI) MatLab (The Mathworks Inc.), NMR acquisition: Topspin 3.0 (Bruker Biospin), SpinSolve + SpinSolve Expert 1.25 (Magritek)
Data analysis	ChemDraw 20.0 (PerkinElmer), GraphPad Prism 7 (GraphPad Software), MatLab 2020 (The Mathworks Inc.), Mestrelab MNova 14.2.0 (Mestrelab), Origin 2018/2021 (OriginLab), QuPath 0.3.2 (Peter Bankhead), BioRender (BioRender)

For manuscripts utilizing custom algorithms or software that are central to the research but not yet described in published literature, software must be made available to editors and reviewers. We strongly encourage code deposition in a community repository (e.g. GitHub). See the Nature Portfolio guidelines for submitting code & software for further information.

Data

Policy information about availability of data

All manuscripts must include a data availability statement. This statement should provide the following information, where applicable:

- Accession codes, unique identifiers, or web links for publicly available datasets
- A description of any restrictions on data availability
- For clinical datasets or third party data, please ensure that the statement adheres to our policy

The raw data generated in this study have been deposited to mediaTUM: https://mediatum.ub.tum.de/1715708 (DOI: 10.14459/2023mp1715708). Source data are provided with this paper.

Research involving human participants, their data, or biological material

Policy information about studies with human participants or human data. See also policy information about sex, gender (identity/presentation), and sexual orientation and race, ethnicity and racism.

Reporting on sex and gender	For studies with healthy animals female animals were used as no sex related differences are expected for a method validation. For the pathological study with MENX rats, both female and male rats were included in the study.
Reporting on race, ethnicity, or other socially relevant groupings	n/a
Population characteristics	n/a
Recruitment	n/a
Ethics oversight	n/a

Note that full information on the approval of the study protocol must also be provided in the manuscript.

Field-specific reporting

Please select the one below that is the best fit for your research. If you are not sure, read the appropriate sections before making your selection.

☒ Life sciences ☐ Behavioural & social sciences ☐ Ecological, evolutionary & environmental sciences

For a reference copy of the document with all sections, see nature.com/documents/nr-reporting-summary-flat.pdf

Life sciences study design

All studies must disclose on these points even when the disclosure is negative.

Sample size	No sample size calculation was performed, sample sizes were chosen based on progress of methods development for the study with healthy animals and with n=4 (both female and male) for the pathological model as a pilot study.
Data exclusions	No data were excluded from the analysis.
Replication	The findings in this study were reproducible, also confirmed by the application of the novel imaging agent in different models (tumor, kidney, hydronephrosis). Experiments were independently replicated for pH imaging in healthy kidneys (n = 10), simultaneous imaging of perfusion and pH in healthy kidneys (n = 9) and hydronephrotic kidneys (n = 8) and EL4-tumor bearing mice (n = 12).
Randomization	No longitudinal studies were performed. All animals were supplied from Charles RIver without any preselection. Imaging on healthy animals was performed in arbitrary order on different days. Tumor implantation was performed in arbitrary order. Hydronephrosis models develop pathologies with 100% probability and imaging was performed in arbitrary order. No treatments or longitudinal studies were conducted. For hydronephrosis models, healthy animals of similar strain were used as controls.
Blinding	Investigators were not blinded as no treatment groups or longitudinal studies were performed. Tumor implanted animals and hydronephrosis models developed tumors and hydronephrotic kidneys with 100% probability. No references were available for pH imaging in hydronephrosis models which could skew expectation outcomes.

Reporting for specific materials, systems and methods

We require information from authors about some types of materials, experimental systems and methods used in many studies. Here, indicate whether each material, system or method listed is relevant to your study. If you are not sure if a list item applies to your research, read the appropriate section before selecting a response.

Materials & experimental systems

n/a	Involved in the study	
☒	☐	Antibodies
☐	☒	Eukaryotic cell lines
☒	☐	Palaeontology and archaeology
☐	☒	Animals and other organisms
☒	☐	Clinical data
☒	☐	Dual use research of concern
☒	☐	Plants

Methods

n/a	Involved in the study	
☒	☐	ChIP-seq
☒	☐	Flow cytometry
☐	☒	MRI-based neuroimaging

Eukaryotic cell lines

Policy information about cell lines and Sex and Gender in Research

Cell line source(s)	EL4 cells were bought from ATCC (https://www.atcc.org/products/tib-39, accessed on 09.07.2023). HeLa cells were bought from ATCC (https://www.atcc.org/products/ccl-2, accessed on 09.07.2023).
Authentication	Only cells with passage number < 10 number were used for cytotoxicity experiments and tumor cell implantation.
Mycoplasma contamination	Only cells with passage number < 10 number were used for cytotoxicity experiments and tumor cell implantation which minimizes the risk for mycoplasma contaminations. Explicit testing immediately before or after cytotoxicity experiments and tumor cell implantation was not performed.
Commonly misidentified lines (See ICLAC register)	For EL4 and HeLa cell lines, no commonly misidentified lines are reported according to the ICLAC register version 12 (16 january 2023).

Animals and other research organisms

Policy information about studies involving animals; ARRIVE guidelines recommended for reporting animal research, and Sex and Gender in Research

Laboratory animals	For pH imaging of healthy kidney, 10 healthy female Wistar rats (Charles River, 7-11 weeks) were used. For pH imaging of subcutaneous EL4 tumors, 9 female C57BL/6 mice (Charles River, 8 weeks) were used. For simultaneous imaging of pH and perfusion, nine female Wistar rats (Charles River, 9-19 weeks) were used. For the hydronephrosis study 4 male and 4 female MENX were used (in-house bred, 8-9 months). Animals were housed in specialized animal housing facilities which are kept at 21-22°C ambient temperature and 40-60% relative humidity and are operated with a 12-hour light and 12-hour dark cycle with dusk transition periods.
Wild animals	The study did not involve wild animals.
Reporting on sex	Sex was considered for the hydronephrosis model and both male and female MENX rats were used.
Field-collected samples	The study did not involve samples collected from the field.
Ethics oversight	All animal experiments were performed in accordance with pertinent laws and regulations and approved by an ethical review board (Regierung von Oberbayern, ROB-55.2-2532.Vet_02-17-177, ROB-55.2-2532.Vet_02-16-117 and ROB-55.2-2532.Vet_02-15-11).

Note that full information on the approval of the study protocol must also be provided in the manuscript.

Magnetic resonance imaging

Experimental design

Design type	n/a
Design specifications	n/a
Behavioral performance measures	n/a

Acquisition

Imaging type(s)	functional, structural, perfusion
Field strength	7T
Sequence & imaging parameters	For phantom imaging, a 72 mm dual-tuned 1H / 13C volume resonator for signal transmission and a 30 mm single channel flexible surface coil (Rapid Biomedical, Germany) attached to the bottom of the water bath for signal acquisition was used. For imaging of rats, the same coil as for phantom imaging was used for transmission and a two-channel flexible coil 13C receive array (Rapid Biomedical, Germany) centred on the back above the rat kidneys was used for signal reception. For imaging of subcutaneous tumors in mice, a 31 mm dual tuned 1H / 13C coil was used for signal transmission and detection. In vivo T1 relaxation time constants for 13C-labels of Z-OMPD was measured using a slice selective excitation in axial orientation covering both kidneys with FA = 10 °, TR = 3 s, 100 repetitions, slice thickness = 15 mm, excitation bandwidth = 16 kHz, receive bandwidth (BW) = 3.2 kHz, 512 spectral points, total scan time = 300 s. Spectra were zero filled, line broadened by 20 Hz in MATLAB (The Mathworks Inc., Natick, USA) and fitted with a three parameter mono exponential decay curve in Origin (OriginLab, Northampton, USA). For hyperpolarized MRI, for localization and co-registration of kidneys or tumors, T2-weighted 1H anatomical images were acquired using RARE with typical parameters: Echo time (TE) = 40 ms, TR = 4000 ms, in-plane resolution = 0.3 mm^2, 1 mm slice thickness. pH imaging in phantom, rats, and mice was performed using a free induction decay chemical shift imaging sequence. Acquisition started after phantom placement in the bore or 9 s post end of injection for in vivo experiments. For buffer phantom imaging, CSI used FA = 10°, TR = 83.1 ms, field of view (FOV) = 60 x 36 mm^2, slice thickness = 6 mm, resolution 3 x 3 x 6 mm^3, BW = 3.2 kHz, 256 spectral points, total scan time = 20 s. For blood phantom imaging, parameters were the same apart from FOV = 64 x 64 mm^2, 5 mm slice thickness, resolution 4 x 4 x 5 mm^3, total scan time 21.4 s. For pH

imaging of kidneys in healthy rats, CSI parameters were FA = 10°, TR = 83.1 ms, FOV = 54 x 42 mm², matrix size = 18 x 14, slice thickness = 5 mm, resolution 3 x 3 x 5 mm³, BW = 3.2 kHz, spectral points = 256, total scan time = 21.0 s. For high resolution pH imaging in subcutaneous EL4, CSI used FA = 15°, TR = 83.1 ms, FOV = 22.4 x 28 mm², slice thickness = 3 mm, resolution 1.4 x 1.4 x 3 mm³, BW = 3.2 kHz, 256 spectral points, total scan time = 26.6 s. For high resolution pH imaging in healthy rat kidneys, acquisition parameters were FA = 2.5°, TR = 83.1 ms, FOV = 60 x 54 mm², slice thickness = 5 mm, resolution 2 x 2 x 5 mm³, BW = 3.2 kHz, 256 spectral points, total scan time = 67 s.
To image perfusion, the C1-label of Z-OMPD, exhibiting only a weak pH-sensitive change in chemical shift, was excited using a slightly off-resonant narrow bandwidth excitation, to avoid excitation of the C5-resonance and the co-injected [13C]urea peak. Both substances were injected at equal concentrations. Excitation and signal readout were performed with a 3D bSSFP sequence (frequency response profile Fig. S9) starting with start of injection. This acquisition was alternating with a narrow-bandwidth bSSFP acquisition using the same parameters and the excitation and readout frequency being placed on the [13C]urea resonance, thereby generating a time series of 3D images for both the C1-label of Z-OMPD and [13C]urea to allow a direct comparison of perfusion imaging using Z-OMPD and [13C]urea within the same injection. This compound-alternating perfusion imaging acquisition lasting 32 s was followed immediately by a 2D CSI acquisition to image pH. For perfusion imaging of the C1-resonance only, a modified sequence was developed with a larger excitation bandwidth to improve robustness under shim variations. Prior to hyperpolarized acquisitions, transmit B1 was calibrated using a 8 M [13C]urea phantom doped with 0.5 mM DOTA placed next to the coil and region of interest and excitation pulses of varying coil power were used to detect the 180° transient. Accurate frequency calibration for the narrow-bandwidth excitation was performed by first measuring the 13C frequency of the urea phantom or of a Z-OMPD phantom in case of non-alternating acquisitions. This frequency was then fine-tuned to frequency within the kidney region due to shim variations between the phantom and the kidney regions. For this purpose, relative frequency differences of the water peak from a 1H press acquisition on the phantom and the kidney region were acquired. 3D bSSFP parameters for alternating perfusion imaging were FA = 12°, excitation bandwidth = 180 Hz, TE = 6.4 ms, TR = 12.8 ms, readout bandwidth = 51 kHz, FOV = 60 x 54 x 30 mm³, matrix size = 20 x 18 x 10, resolution 3 mm³ isotropic, number of frames per compound = 7, frame scan time per compound = 2.3 s, temporal resolution 4.6 s for each compound, total scan time 32.4 s. For imaging of the Z-OMPD C1-resonance only without co-injected [13C]urea, the acquisition used FA = 12°, excitation bandwidth = 240 Hz, TE = 5 ms, TR = 10 ms, readout bandwidth = 39 kHz, number of frames = 9, frame scan time = 1.8 s, temporal resolution 3.6 s. total scan time 32.5 s. The CSI acquisition following bSSFP used FA = 10°, TR = 83.1 ms, FOV = 60 x 54 mm², slice thickness = 5 mm, resolution 3 x 3 x 5 mm³, receive bandwidth = 3.2 kHz, 256 spectral points, total scan time = 29.9 s. Hydronephrosis models were imaged with bSSFP parameters FA = 12°, excitation bandwidth = 240 Hz, TE = 5 ms, TR = 10 ms, readout bandwidth = 32.9 kHz, FOV = 64 x 64 x 40 mm³, matrix size = 16 x 16 x 10, resolution 4 mm³ isotropic, number of frames = 10, frame scan time = 1.61 s, temporal resolution 3.22 s. total scan time 32.2 s and CSI parameters FA = 10°, TR = 83.1 ms, FOV = 64 x 56 mm², slice thickness = 4 mm, resolution 4 mm³ isotropic, receive bandwidth = 3.2 kHz, 256 spectral points, total scan time = 18.6 s.

Area of acquisition: either tumor or abdominal regions

Diffusion MRI: ☒ Used ☐ Not used

Parameters: Imaging of diffusivity used a standard Bruker DtiEpi-Sequence with echoplanar imaging readout, 16 b-values (10, 20, 40, 60, 80, 100, 200, 300, 400, 500, 600, 700, 800, 1000, 1200, 1500), monopolar diffusion encoding and typical parameters TE = 25 ms, TR = 5000 ms, readout bandwidth = 100 kHz, FOV = 60 x 66 x 30 mm³, resolution 1 mm³ isotropic, 6 repetitions, total scan time 8 min 30s. ADC values were fitted pixelwise to the signal decay curves with increasing b-values using a mono-exponential function with offset.

Preprocessing

Preprocessing software: n/a

Normalization: n/a

Normalization template: n/a

Noise and artifact removal: n/a

Volume censoring: n/a

Statistical modeling & inference

Model type and settings: n/a

Effect(s) tested: n/a

Specify type of analysis: ☐ Whole brain ☐ ROI-based ☐ Both

Statistic type for inference: n/a

(See Eklund et al. 2016)

Correction: n/a

Models & analysis

n/a	Involved in the study
☒	☐ Functional and/or effective connectivity
☒	☐ Graph analysis
☒	☐ Multivariate modeling or predictive analysis
